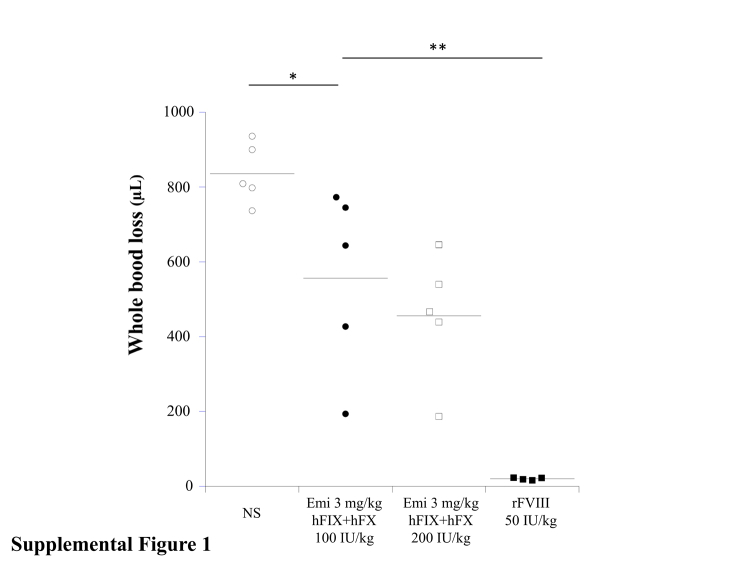# Augmented emicizumab-driven coagulation potential in hemophilia A state by *in vitro* and *in vivo* supplementation of combined factors IX and X

**DOI:** 10.1016/j.rpth.2025.103329

**Published:** 2025-12-30

**Authors:** Mitsumasa Osuna, Yuto Nakajima, Eisuke Takami, Hirotoshi Nakano, Keiji Nogami

**Affiliations:** 1 Department of Pediatrics, Nara Medical University, Kashihara, Nara, Japan; 2 Medical Affairs Section, KM Biologics Co, Ltd, Kumamoto, Japan

**Keywords:** antibodies, bispecific, factor IX, factor X, hemophilia A, hemostasis

## Abstract

**Background:**

Persons with hemophilia A (HA) and inhibitors undergoing emicizumab prophylaxis require bypassing agents when breakthrough bleeding occurs. Recent studies have demonstrated that either factor (F)IX or FX supplementation can improve coagulation potential of emicizumab-treated persons with HA and inhibitors.

**Objectives:**

This study assessed the effect of combined supplementation with FIX and FX on the coagulation potential in emicizumab-treated HA state.

**Methods:**

FVIII-deficient plasmas were spiked with emicizumab (50 μg/mL), FX (100 IU/dL), and various FIX levels (100-1600 IU/dL). Plasmas from emicizumab-treated persons with HA and inhibitors were also added with FIX/FX (100 IU/dL each). Coagulation potential was assessed by maximum coagulation velocity (Ad|min1|) using tissue factor (TF)/ellagic acid–triggered clot waveform analysis and peak thrombin (PeakTh) using TF-triggered thrombin generation assay. For *in vivo* method, emicizumab (3 mg/kg) and human (h)FIX/hFX (100 IU/kg each) were intravenously administered to HA mice (emicizumab-HA mice). Coagulation potentials in these mice with or without additional hFIX/hFX (100 IU/dL each) were assessed by clotting time plus clot formation time (CT + CFT) and blood loss using rotational thromboelastometry and tail-clip assay.

**Results:**

Ad|min1| and PeakTh in FVIII-deficient plasmas with emicizumab, FIX, and FX increased in FIX dose dependently. Addition of FIX and FX (100 IU/dL each) to emicizumab-supplemented FVIII-deficient plasma and plasma of emicizumab-treated persons with HA and inhibitor improved both parameters to normal levels. CT+CFT and blood loss in emicizumab-HA mice with additional hFIX/hFX (100 IU/dL each) administration were significantly shorter and decreased than those in emicizumab-HA mice. The thrombotic markers largely did not change.

**Conclusion:**

Combined FIX and FX supplementation could enhance coagulation potential in emicizumab-treated persons with HA and inhibitors.

## Essentials

• Effect of additional factor (F)IX and FX on emicizumab-driven hemostasis is unclear.
• Additional FIX and FX improved coagulation function in hemophilia A (HA) plasma spiked with emicizumab.
• Administration of additional human FIX and FX enhanced emicizumab-mediated hemostasis in HA mice.
• FIX and FX may further improve emicizumab-driven hemostasis in HA.

## Graphical abstract

**Figure:**
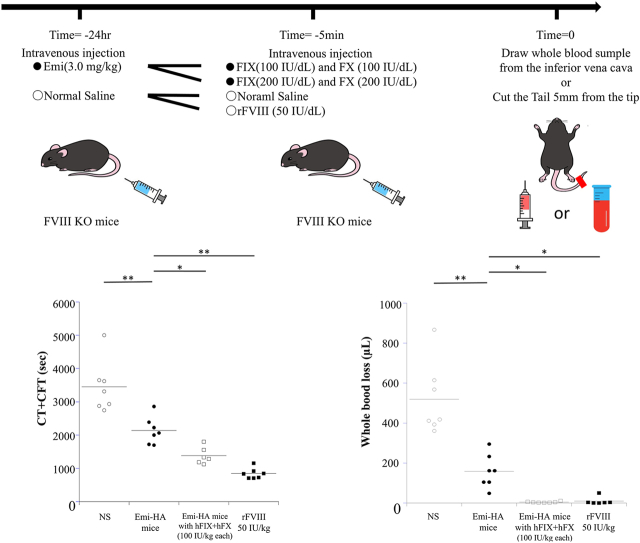

## 1 Introduction

Hemophilia A (HA) is a common hereditary bleeding disorder caused by a deficiency or dysfunction in coagulation factor (F)VIII. Approximately 20% to 30% of people with severe HA develop anti-FVIII alloantibodies (inhibitors) following repeated administration of FVIII products [1]. The presence of FVIII inhibitors complicates the clinical management of these patients [2]. For hemostatic treatment in such cases, bypassing agents (BPAs), such as recombinant activated FVII (rFVIIa; NovoSeven; Novo Nordisk) and activated prothrombin complex concentrates (APCCs; FEIBA; Takeda Japan), are commonly used [3]. Additionally, a plasma-derived mixture of FVIIa and FX (pd-FVIIa/FX; Byclot; KM Biologics Co) is available in Japan for on-demand and prophylactic treatment of patients with inhibitors [4,5]. Although these BPAs are effective in enhancing hemostasis, their clinical efficacy may vary among individuals due to multiple contributing factors [6,7].

Emicizumab (Hemlibra; Chugai Pharmaceutical Co) is a recombinant, humanized, bispecific monoclonal antibody that binds to FIX/FIXa and FX/FXa and mimics the cofactor activity of FVIIIa in the tenase complex [8,9]. Prophylactic subcutaneous administration of emicizumab has been confirmed to significantly reduce bleeding episodes in persons with severe HA, irrespective of the presence of FVIII inhibitors [10,11]. The clinically therapeutic concentration of emicizumab (50 μg/mL) is estimated to be equivalent of only 10 to 20 IU/dL of FVIII [12,13], and concomitant administration of BPAs remains necessary for the management of breakthrough bleeding or during major surgical interventions especially in persons with HA and inhibitors [12]. However, thrombotic events have been reported in some persons with HA receiving concomitant therapy of emicizumab with APCC [11], leading to the recommendation of rFVIIa as the preferred first-line hemostatic agent in such settings. It is important to note that rFVIIa does not always provide sufficient hemostatic efficacy and that its repeated administration diminishes its effectiveness [14,15]. Alternative therapeutic options that ensure both safety and efficacy may clinically benefit persons with HA and inhibitors undergoing emicizumab therapy.

A recent study showed that emicizumab enhanced the coagulation potential of plasma from selected persons with hemophilia B [16]. In addition, coagulation function in plasma from emicizumab-treated persons with HA revealed a FIX dose-dependent improvement in the thrombin generation assay (TGA) [17]. The addition of exogenous FIX may enhance the hemostatic efficacy of emicizumab, whereas excessively elevated FIX activity, such as that observed in individuals with the FIX-Padua variant, is associated with an increase in thrombotic risk [18].

Moreover, we have demonstrated that additional FX supplementation to the plasma from emicizumab-treated persons with HA and FX administration into emicizumab-treated HA mice improved coagulation potentials in a FX dose–dependent manner [19]. Notably, thrombotic events were observed after rFVIIa administration, potentially because excessive generation of FXa facilitates thrombosis [20]. Therefore, these findings suggest that although supplementation with either FIX or FX alone enables the restoration of hemostatic function in the HA state with emicizumab, higher doses may potentially increase the thrombotic risk.

In this context, we hypothesized that combined supplementation with low concentrations of exogenous FIX and FX may enhance the coagulation potential in persons with HA receiving emicizumab without increasing the thrombotic risk. A surface plasmon resonance–based assay revealed that the calculated dissociation constants (*K*_d_) for the interaction with emicizumab to FIX and FX are 1.58 and 1.85 μM, respectively [21], indicating that the physiological plasma concentrations of FIX (∼87 nM) and FX (∼130 nM) [22,23] seems to be insufficient to substantially impact the hemostatic function of emicizumab. Based on these findings, we assumed that co-administration of FIX and FX *in vivo* may potentiate emicizumab activity and provide hemostatic benefit when breakthrough bleeding occurs in emicizumab-treated persons with HA with inhibitors. The present study was designed to investigate the global coagulation potential in the HA state under emicizumab-treated conditions in the presence of additional combined FIX and FX using *in vitro* and *in vivo* experimental models.

## 2 Methods

This study was approved by the Medical Research Ethics Committee of Nara Medical University (No. 2863). Blood samples were collected after obtaining informed consent in accordance with the ethical guidelines of our university. All animal experiments were approved by the Animal Care and Use Committee of Nara Medical University (No. 13231), and all procedures followed the Policy on the Care and Use of Laboratory Animals of Nara Medical University.

### 2.1 Reagents

Emicizumab [8,9], rFVIIa (NovoSeven), and rFVIII (rurioctocog alpha) were obtained from Chugai Pharmaceutical Co, Novo Nordisk, and Takeda Japan, respectively. Highly purified human (h)FIX and hFX obtained by immunoaffinity chromatography were provided by KM Biologics Co Ltd. Recombinant human tissue factor (TF) (Innovin; Dade), ellagic acid (Sysmex Corporation), plasma from persons with HA without inhibitors (George-King), thrombin-specific fluorogenic substrate FluCa kit and thrombin calibrator (Thrombinoscope BV), mouse thrombin–antithrombin (TAT) complex ELISA kit (ab137994; Abcam), and mouse D-dimer ELISA kit (EEL094; Thermo Fisher Scientific) were purchased from the indicated vendors. Prothrombin time (PT) and activated partial thromboplastin time (APTT) reagents (Revohem PT and Thrombocheck APTT-SLA; Sysmex) were used as TF and ellagic acid sources, respectively. PL vesicles containing 10% phosphatidylserine, 60% phosphatidylcholine, and 30% phosphatidylethanolamine were prepared [24].

### 2.2 Patient profiles

Blood samples were prepared from 3 patients with congenital HA and inhibitors receiving emicizumab prophylaxis. The dose regimen during the maintenance period in cases 1 and 2 was 3 mg/kg subcutaneously once every 2 weeks, and that in case 3 was 1.5 mg/kg subcutaneously once a week. Briefly, the inhibitor titers measured using the Bethesda assay prior to the introduction of emicizumab in cases 1 to 3 were 48.1, 5.36, and 22.1 BU/mL, respectively. The FIX:C and FX:C measured by 1-stage clotting assays were 69.5 and 84.0 IU/dL (case 1), 100.8 and 129.5 IU/dL (case 2), and 61.9 and 91.6 IU/dL (case 3), respectively. The emicizumab concentration in patients’ plasma was measured by a modified FVIII 1-stage clotting assay as previously reported [12].

### 2.3 Global coagulation assay

#### 2.3.1 Clot waveform analysis

Clot waveform analysis (CWA) was performed using a CS-2400i instrument (Sysmex) with a trigger mixture comprising TF and ellagic acid [25]. Briefly, plasma samples (50 μL) were preincubated for 3 minutes with PT and APTT trigger reagents (50 μL; PT/APTT/buffer = 1/15/135 [v/v/v]), prior to the addition of 25 mM CaCl_2_ (50 μL) to initiate coagulation. The automated coagulation analyzer detected the transmittance of the light intensity, and the clot waveforms were processed using a commercial kinetic algorithm. Further, |min1| was calculated as the minimum value of the first derivatives of transmittance, reflecting the maximum coagulation velocity achieved. The minimum transmittance (0%) was determined immediately after the coagulation phase, and the adjusted|min1| (Ad|min1|) was defined as the maximum coagulation velocity obtained from the first derivatives of the adjusted waveform [25]. The mean Ad|min1| value obtained from healthy individuals was 7.2 ± 0.6 [26].

#### 2.3.2 Thrombin generation assays

TGA was performed as previously described [27]. Briefly, plasma samples (80 μL) were preincubated for 10 minutes with 20 μL of a trigger reagent containing TF and PL (final concentration: 1.0 pM and 4 μM, respectively). After adding 20 μL of reagent containing CaCl_2_ and fluorogenic substrate (final concentration: 16.7 and 2.5 mM, respectively), the development of fluorescent signals was monitored using a Fluoroskan Ascent microplate reader (Thermo Fisher Scientific). Data analyses were performed using the manufacturer’s software to derive standard peak thrombin (PeakTh) and endogenous thrombin potential (ETP) parameters. The PeakTh and ETP values obtained from 15 healthy individuals were 215 ± 37 nM and 2882 ± 306 nM × minute, respectively [28].

#### 2.3.3 Rotational thromboelastometry

Reportedly, nonactivated thromboelastometry (NATEM) mode of rotational thromboelastometry (ROTEM) reflects the global coagulation function in persons with HA receiving emicizumab [28]. Therefore, this method was adopted using a Whole Blood Hemostasis Analyzer (Pentapharm). The citrated whole blood samples (280 μL) were incubated for 30 minutes at room temperature prior to the addition 20 μL CaCl_2_ (final concentration: 12.5 mM) to initiate coagulation. Clot formation was evaluated using the following parameters: clotting time (CT; the period until reaching a 2-mm amplitude), clot formation time (CFT; the period until reaching a 20-mm amplitude), and CT+CFT. ROTEM parameters reflect coagulation function in persons with HA receiving emicizumab [29]. The IQRs of CT and CT + CFT in 20 healthy individuals were defined as 802 to 1041 seconds and 1103 to 1413 seconds, respectively [29].

### 2.4 The spiking protocol involving the addition of exogenous FIX and FX to FVIII-deficient plasma supplemented with emicizumab or rFVIII or to plasmas from emicizumab-treated persons with HA and inhibitors

Increasing concentrations of hFIX (up to 1600 IU/dL) and/or hFX (100 IU/dL) were added to FVIII-deficient plasma (the mean FIX and FX activities were 95 and 121 IU/dL, respectively) in the presence or absence of emicizumab (50 μg/mL) or rFVIII (10 IU/dL), and their coagulation potential was evaluated by CWA and TGA. Regarding ROTEM analysis, the exogenous FIX and FX (100 IU/dL each) were added to whole blood samples from 3 emicizumab-treated persons with HA and inhibitors. Furthermore, the rFVIIa (2.2 μg/mL) or exogenous FIX/FX (100 IU/dL each) was added to plasma samples from 3 emicizumab-treated persons with HA with inhibitors and their coagulation potential were evaluated by CWA and TGA. As for healthy controls of CWA and TGA, their coagulation potential was examined in recent studies [26,28]. We previously demonstrated that CV intra-assay in TF-triggered TGA was <10%, and that in modified CWA was <5% [25,30]. Thus, data from the present study were compared with those of healthy controls from the previous studies.

### 2.5 Preparation of HA mice

HA mice with the targeted destruction of exon 16 on a pure 129 C57BL/6 background were kindly gifted by Dr Yoichi Sakata (Jichi Medical University, Shimotsuke, Japan) and backcrossed with C57BL/6 mice (CLEA Japan, Tokyo, Japan) as previously described [19]. Male and female mice were aged 8 to 12 weeks (body weight: 20-28 g). All the animals were maintained under specific pathogen-free conditions.

### 2.6 *In vivo* hemostatic assay on HA mice treated with emicizumab in the presence of hFIX and hFX

#### 2.6.1 Tail-clip assay

The tail-clip assay was performed as previously described [19]. Briefly, HA mice were anesthetized with a mixture of medetomidine, midazolam, and butorphanol and then placed on a hot plate (Tokyo Garasu Kikai) at 37 °C for at least 10 minutes. HA mice were administered emicizumab (3 mg/kg) intravenously (24 hours before tail amputation). For the second administration, both hFIX and hFX (100 or 200 IU/kg each) were administered 5 minutes before tail amputation [19]. HA mice treated with emicizumab and hFIX/hFX (100 IU/kg each) were termed emicizumab-HA mice [19], and those treated with hFIX/hFX (200 IU/kg each) were termed emicizumab-HA mice with additional hFIX/hFX (100 IU/kg each). The tail was cut 5 mm from the tip 5 minutes after the second administration and immediately placed in a conical tube containing 10 mL of saline (prewarmed to 37 °C). The tails of HA mice administered with rFVIII (50 IU/kg) were also cut 5 mm from the tip, as in other HA mice. The tail was immediately immersed in a 50-mL Falcon tube containing isotonic saline prewarmed in a water bath to 37 °C. The position of the tail was vertical with the tip positioned about 1 to 2 cm below the body horizon for 10 or 30 minutes as previously described [31]. The weight of the Falcon tube containing isotonic saline was recorded prior to the experiment, and blood loss was calculated by subtracting this prerecorded weight from the weight of the Falcon tube after the experiment.

#### 2.6.2 ROTEM assay

NATEM was used to assess the coagulation potential in HA mice administered with emicizumab and both hFIX and hFX using a whole blood hemostasis analyzer. Briefly, isoflurane-anesthetized HA mice were administered emicizumab (3 mg/kg) intravenously (24 hours before blood draw). A second administration of hFIX/hFX (100 or 200 IU/kg each) was administered 5 minutes before the blood draw (termed emicizumab-HA mice and emicizumab-HA mice with additional hFIX/hFX [100 IU/kg each]). Whole blood samples were drawn from the inferior vena cava 5 minutes after the second administration. Whole blood samples from HA mice intravenously administered rFVIII (50 IU/kg) were also drawn from the inferior vena cava. Blood samples were analyzed using ROTEM. Coagulation potential was evaluated using parameters of CT, CFT, α-angle (the angle of tangent between 0 mm and the curve when the clot firmness is 20 mm), and maximum clot firmness.

### 2.7 D-dimer and TAT complexes in HA mice receiving emicizumab and additional hFIX and hFX

The D-dimer and TAT complex levels were measured in HA mice administered emicizumab together with hFIX (100 IU/kg) and hFX (100 IU/kg), as well as in HA mice administered emicizumab together with hFIX (200 IU/kg) and hFX (200 IU/kg). Briefly, isoflurane-anesthetized HA mice were first infused with emicizumab (3 mg/kg) intravenously (24 hours before blood draw). A second injection containing hFIX (100 or 200 IU/kg) and hFX (100 or 200 IU/kg) was given 5 minutes before the blood draw. The whole blood samples were drawn from inferior vena cava and collected into tubes containing 3.2% sodium citrate at a 9:1 ratio 5 minutes after the second injection. The D-dimer and TAT complexes in those blood samples were measured, according to the manufacturer’s instructions.

### 2.8 Simulated the concentrations of FIX-emicizumab-FX ternary complexes in HA plasma containing emicizumab

We simulated equilibrium states in HA plasma containing emicizumab (50 μg/mL; approximately 340 nM), as previously described [21]. The concentrations of the FIX–emicizumab–FX ternary complex were calculated in HA plasma containing emicizumab with additional FIX/FX (100 IU/dL each), as well as in HA plasma containing emicizumab alone.

### 2.9 Data analysis

Mean values and SDs of all *in vitro* experiments of FVIII-deficient plasmas are shown. Significant differences in *in vitro* experiments of FVIII-deficient plasmas and *in vivo* experiments were determined using Dunnett multiple comparison test. Comparison of D-dimer and TAT levels in HA mice was performed using the Dunnett test. Data analysis was performed using the Kaleida Graph software 5.0 (Synergy). Statistical significance was set at *P* < .05.

## 3 Results

### 3.1 Coagulation potential on the addition of exogenous FIX and FX to FVIII-deficient plasmas supplemented with emicizumab

**Figure 1:**
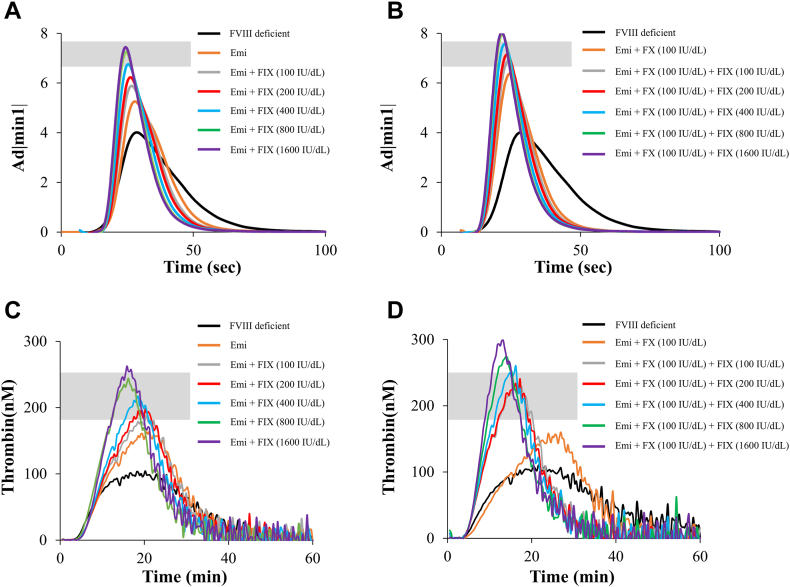
*In vitro* coagulation potentials determined by global coagulation assays in the presence of emicizumab and various amounts of factor (F)IX in FVIII-deficient plasmas with or without FX. FVIII-deficient plasma with or without FX were mixed with various amounts of FIX (up to 1600 IU/dL) and emicizumab (50 μg/mL) *in vitro*, prior to the measurement by clot waveform assay and thrombin generation assay as described in the Methods section: the clot waveform curves for FVIII-deficient plasma with emicizumab (A) and various amounts of FIX and/or FX (100 IU/dL) (B). Thrombin generation curves in FVIII-deficient plasma with emicizumab (C) and various amounts of FIX and/or FX (100 IU/dL) (D). Two different batches of FVIII-deficient plasma were examined twice for each batch. The gray bars represent the reference ranges obtained from healthy individuals. Representative clot waveform curves (A, B) and thrombin generation curves (C, D) are illustrated. The lines shown are follows: black, FVIII-deficient plasma (def-p); orange, FVIII def-p + emicizumab (Emi); gray, FVIII def-p + Emi + FIX 100 IU/dL; red, FVIII def-p + Emi + FIX 200 IU/dL; blue, FVIII def-p + Emi + FIX 400 IU/dL; green, FVIII def-p + Emi + FIX 800 IU/dL; purple, FVIII def-p + Emi + FIX 1600 IU/dL.

First, coagulation potentials in FVIII-deficient plasma supplemented with emicizumab (50 μg/mL), hFX (100 IU/dL) and increasing concentrations of hFIX (up to 1,600 IU/dL) were evaluated by global coagulation assays such as CWA and TGA as described in the Methods section. Representative curves are shown in Figure 1A–D, and the obtained parameters are summarized in Table 1.

**Table 1:** *In vitro* parameters determined by global coagulation assays in FVIII-deficient plasmas mixed with rFVIII or emicizumab after addition of various amounts of exogenous FIX and FX 100 IU/dL

Parameters		FVIII-def	No FX						FX 100				
			No FIX	FIX 100	FIX 200	FIX 400	FIX 800	FIX 1600	FIX 100	FIX 200	FIX 400	FIX 800	FIX 1600
**Emicizumab (50 μg/mL)**													
CWA	Ad|min1|	3.6 ± 0.2∗∗	5.4 ± 0.2	6.1 ± 0.2∗∗	6.4 ± 0.2∗∗	6.8 ± 0.1∗∗	7.2 ± 0.2∗∗	7.3 ± 0.4∗∗	6.8 ± 0.0∗∗	7.1 ± 0.1∗∗	7.5 ± 0.1∗∗	8.0 ± 0.3∗∗	8.2 ± 0.5∗∗
TGA	PeakTh (nM)	103 ± 3∗∗	159 ± 4	180 ± 1	192 ± 0	215 ± 5∗∗	230 ± 8∗∗	240 ± 16∗∗	225 ± 2∗∗	228 ± 1∗∗	241 ± 7∗∗	262 ± 8∗∗	279 ± 15∗∗
	ETP (nM × min)	2784 ± 262	3070 ± 120	3016 ± 244	2997 ± 238	3044 ±161	3060 ± 178	3115 ± 349	3157 ± 347	3036 ± 282	3034 ± 325	3143 ± 168	3224 ± 320
**rFVIII (10 IU/dL)**													
CWA	Ad|min1|	4.1 ± 0.1∗∗	4.5 ± 0.2	5.1 ± 0.0∗∗	5.3 ± 0.1∗∗	5.3 ± 0.2∗∗	5.5 ± 0.1∗∗	5.5 ± 0.0∗∗	5.2 ± 0.1∗∗	5.3 ± 0.2∗∗	5.4 ± 0.1∗∗	5.5 ± 0.1∗∗	5.5 ± 0.1∗∗
TGA	PeakTh (nM)	106 ± 27	136 ± 9	156 ± 1	167 ± 19	170 ± 26	155 ± 11	148 ± 18	199 ± 0	214 ± 2	215 ± 4	245 ± 37∗	235 ± 30∗
	ETP (nM×min)	2624 ± 311	2806 ± 5	3202 ± 196	3307 ± 205	3061 ±; 69	3069 ± 53	2968 ± 81	3251 ± 355	3343 ± 212	3393 ± 161	3407 ± 126	3474 ± 136

Clot waveform assay (CWA) and thrombin generation assay (TGA) in the presence of emicizumab (50 μg/mL) or rFVIII (rurioctocog alfa; 10 IU/dL) and various amounts of exogenous FIX concentrates and with/without FX 100 IU/dL were performed. Two different batches of FVIII-deficient plasma were examined twice for each batch, and the mean and SD of the parameters are shown. CWA and TGA parameters obtained from FVIII-deficient plasmas supplemented with emicizumab alone were used as the control group and were compared with those from FVIII-deficient plasmas spiked with FIX and/or FX in the presence of emicizumab. Similarly, those parameters obtained from FVIII-deficient plasmas spiked with rFVIII alone were used as the control group and were compared with those from FVIII-deficient plasmas mixed with FIX and/or FX in the presence of rFVIII. Significant differences were considered as *P* < .05. The Ad|min1| value obtained from healthy individuals was 7.2 ± 0.6, and the peak thrombin and ETP values obtained from healthy individuals were 215 ± 37 nM and 2882 ± 306 nM × min, respectively.

Ad|min1|, adjusted |min1|; ETP, endogenous thrombin potential; F, factor; FVIII-def, FVIII-deficient plasma; PeakTh, peak thrombin; rFVIII, recombinant FVIII.

*∗P* < .05; ∗∗*P* < .01.

The CWA findings demonstrated that the addition of emicizumab to the FVIII-deficient plasmas increased the Ad|min1| from 3.6 ± 0.2 to 5.4 ± 0.2, and the further addition of exogenous FIX enhanced this value dose dependently (Figure 1A). The Ad|min1| (6.8 ± 0.1) in FVIII-deficient plasma added with emicizumab and FIX (400 IU/dL) reached within the normal range (7.2 ± 0.6). Similarly, the Ad|min1| (6.8 ± 0.0) in FVIII-deficient plasma added with emicizumab and both FIX and FX (100 IU/dL each) was significantly greater than that in FVIII-deficient plasma added with emicizumab alone and within the normal range (Figure 1B). However, in the absence of emicizumab, the addition of exogenous FIX and FX had little effect on Ad|min1| (Supplementary Table 1). The addition of exogenous FIX and FX to FVIII-deficient plasma mixed with rFVIII (10 IU/dL) significantly enhanced Ad|min1|; however, the magnitude of this increase was smaller than that observed with emicizumab (Table 1).

The PeakTh obtained by TGA in FVIII-deficient plasma in the presence of emicizumab alone was greater than that in its absence (159 ± 4 and 103 ± 3 nM, respectively). PeakTh in the presence of emicizumab and various amounts of exogenous FIX increased in FIX dose–dependent manner (Figure 1C). The addition of emicizumab and 100 IU/dL FIX resulted in a PeakTh close to the normal range (180 ± 1 and 215 ± 37 nM, respectively). Moreover, the PeakTh (225 ± 2 nM) in FVIII-deficient plasma with emicizumab and FIX/FX (100 IU/dL each) was significantly greater than that in FVIII-deficient plasma with emicizumab alone and reached within the normal range, similar to CWA results (Figure 1D). Additional FIX and FX did not enhance the ETP (Table 1). Regarding Ad|min1| value, the addition of exogenous FIX and FX in the absence of emicizumab did not significantly enhance the TGA results in FVIII-deficient plasma (Supplementary Table 1). In contrast, the addition of higher levels of FIX and FX (800 or 1600 IU/dL) to FVIII-deficient plasma mixed with rFVIII (10 IU/dL) significantly increased the PeakTh value, but the magnitude of this increase was smaller than that observed with emicizumab (Table 1). These findings suggested that although the addition of exogenous FIX and FX (100 IU/dL each) enhanced FVIII-mediated coagulation function, it further augmented the emicizumab-driven coagulation potential in FVIII-deficient plasma.

### 3.2 Coagulation potentials on the addition of FIX and FX to blood samples from emicizumab-treated persons with HA and inhibitors

**Figure 2:**
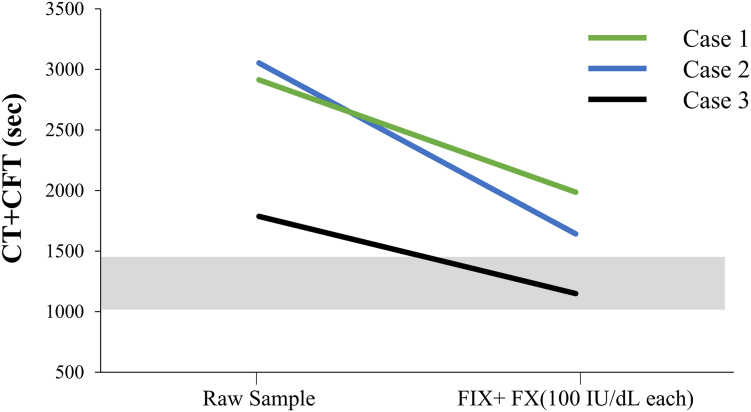
*In vitro* coagulation potential of whole blood in the emicizumab-treated persons with hemophilia A (HA) with inhibitors after the addition of human factor (F)IX (100 IU/kg) and FX (100 IU/kg). Citrated whole blood samples taken from emicizumab-treated persons with HA with inhibitors (*n* = 3) spiked with human (h)FIX (100 IU/dL) and hFX (100 IU/dL) were assessed using rotational thromboelastometry triggered by CaCl_2_ as described in the Methods section. The CT+CFT results obtained using patient samples are derived from a single measurement because the amount of patients’ blood samples was limited. The gray bars represent the reference ranges obtained from healthy individuals. The CT+CFT parameters before and after the spike in FIX/FX are shown. CFT, clot formation time; CT, clotting time; Emi, ecomizumab.

The coagulation potential of exogenous FIX and FX (100 IU/dL each) added to whole blood samples from 3 emicizumab-treated persons with HA with inhibitors (cases 1-3) was assessed using ROTEM. The CT + CFT values of the 3 whole blood samples after the addition of FIX and FX were evidently shorter than those of the raw samples. In particular, in case 3, CT+CFT after the addition of FIX and FX was within the normal range (Figure 2). With regard to the emicizumab concentration in cases 1 to 3, their emicizumab concentration was 41, 32, and 37 μg/mL, respectively.

**Figure 3:**
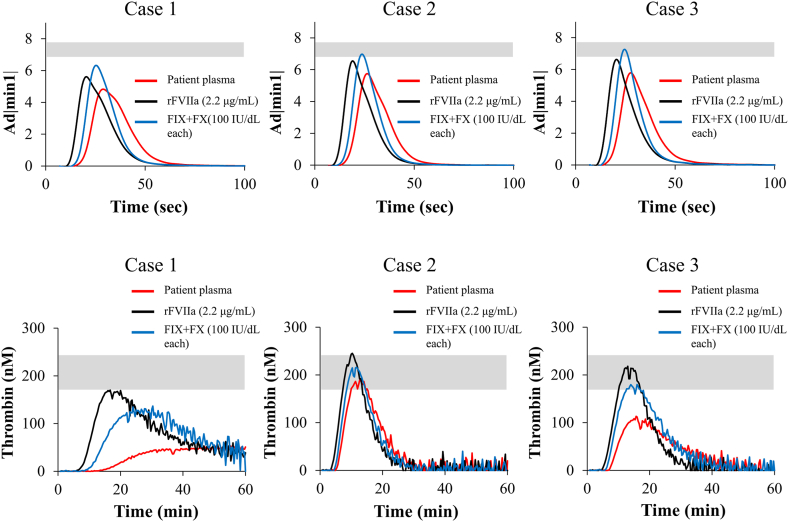
*In vitro* coagulation potential determined by global coagulation assays in the presence of exogenous recombinant (r) factor (F)VIIa or human (h)FIX and FX in plasma samples from emicizumab-treated persons with hemophilia A (HA) and inhibitors. Plasmas from 3 emicizumab-treated persons with HA and inhibitors were mixed with exogenous rFVIIa (2.2 μg/mL; corresponding to 90 μg/kg) or hFIX (100 IU/dL) and hFX (100 IU/dL) *in vitro*, prior to measurement by clot waveform assay (CWA) and thrombin generation assay (TGA) as described in the Methods section. The CWA and TGA results obtained using patient samples were derived from a single measurement because the amount of patients’ blood samples was limited. The gray bars represent the reference ranges obtained from healthy individuals. The clot waveform curves (upper panels) and thrombin generation curves (lower panels) are shown. The lines shown are follows: red, raw sample; black, rFVIIa 2.2 μg/mL; blue, hFIX 100 IU/dL and hFX 100 IU/dL.

According to the clinical guidelines of the on-demand BPA therapy concomitant with emicizumab [29], the use of rFVIIa (2.2 μg/mL in plasma, corresponding to 90 μg/kg) is recommended. Therefore, we assessed the effect of spiking either rFVIIa (2.2 μg/mL) or combined FIX/FX (100 IU/dL each) on global hemostatic potentials, in plasma samples from 3 emicizumab-treated persons with HA and inhibitors. Representative CWA and TGA curves are shown in Figure 3. In the CWA results, the Ad|min1| of the plasma spiked with rFVIIa or FIX/FX (100 IU/dL each) increased compared with that of the raw samples. The Ad|min1| of the plasma spiked with rFVIIa was comparable with that spiked with FIX/FX (100 IU/dL each) and was not beyond the normal range (Figure 3). As for TGA results, the PeakTh of the plasma obtained from case 1 spiking FIX/FX (100 IU/dL each) was slightly lower than that of the corresponding rFVIIa-spiked sample. In cases 2 and 3, the PeakTh values were comparable between the FIX/FX-spiked and rFVIIa-spiked samples and were within the normal range (Figure 3). Overall, these results demonstrated that the combined addition of FIX and FX with emicizumab could improve the coagulation function of persons with HA with inhibitors to an extent comparable with that observed with supplementation of rFVIIa at the recommended dose.

### 3.3 Effects of additional hFIX and hFX in HA mice treated with emicizumab

**Figure 4:**
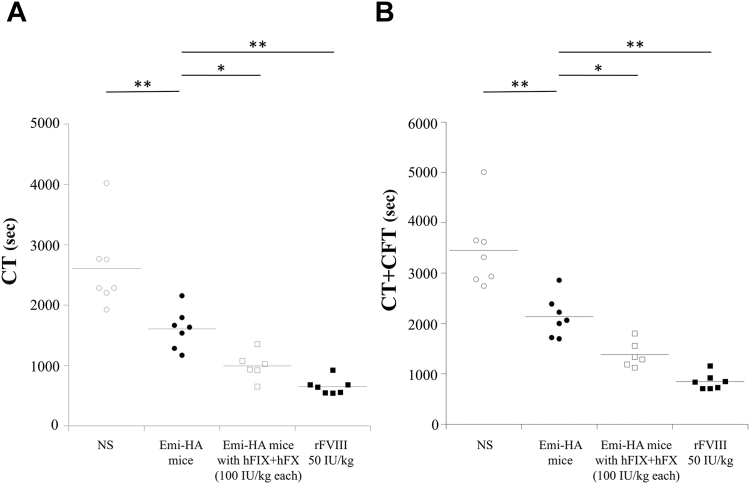
*In vivo* coagulation potential assessed by rotational thromboelastometry (ROTEM) in HA mice administered emicizumab, with 2 types of doses of human (h) factor (F)IX and FX. Emicizumab (3 mg/kg), hFIX (100 IU/kg), and hFX (100IU/kg) were administered to the HA mice (termed Emi-HA mice). Emi-HA mice were administered hFIX and hFX at 100 or 200 IU/kg each. The recombinant (r)FVIII preparation (rurioctocog alfa at 50 IU/kg) was administered to HA mice. CaCl_2_ was added to the citrated whole blood samples for ROTEM analysis. CT (A) and CT+CFT (B) parameters in various HA mice are illustrated. CT (A) and CT+CFT (B) in Emi-HA mice were compared with those in other mice. Statistical analyses between Emi-HA mice (*n* = 7) and other HA mice (*n* = 6-7) were performed using Dunnett multiple comparison test. Significant differences were defined as *P* < .05 (∗*P* < .05; ∗∗*P* < .01). Each data point represents a single mouse. The straight line represents the mean values. CT, clotting time; CFT, clot formation time; Emi, emicizumab; HA, hemophilia A; NS, normal saline.

Recent studies have demonstrated successful experimental methods and data for evaluating the *in vivo* coagulation potential of emicizumab in HA mice [19,32]. In these studies, emicizumab (3 mg/kg), hFIX (100 IU/kg), and hFX (100 IU/kg) were administered intravenously (termed emicizumab-HA mice) based on previous findings [19,32], and our investigation have demonstrated that NATEM could reflect the global coagulation potential in emicizumab-HA mice [19]. Therefore, we compared the coagulation potential of emicizumab-HA mice with or without additional hFIX/hFX (100 IU/kg each) using NATEM analysis (Figure 4). The CT and CT + CFT values in emicizumab-HA mice were significantly shorter than those in naive HA mice. In contrast, those in emicizumab-HA mice with additional hFIX/hFX (100 IU/kg each) and HA mice administered rFVIII (50 IU/kg) were significantly shorter than those in emicizumab-HA mice (Figure 4A, B). The α-angle and maximum clot firmness in all mice were comparable (data not shown). These results demonstrate that the additional administration of hFIX and hFX further augmented the coagulation potential in emicizumab-HA mice.

### 3.4 Tail-clip assay by additional hFIX/hFX supplementation in emicizumab-HA mice

**Figure 5:**
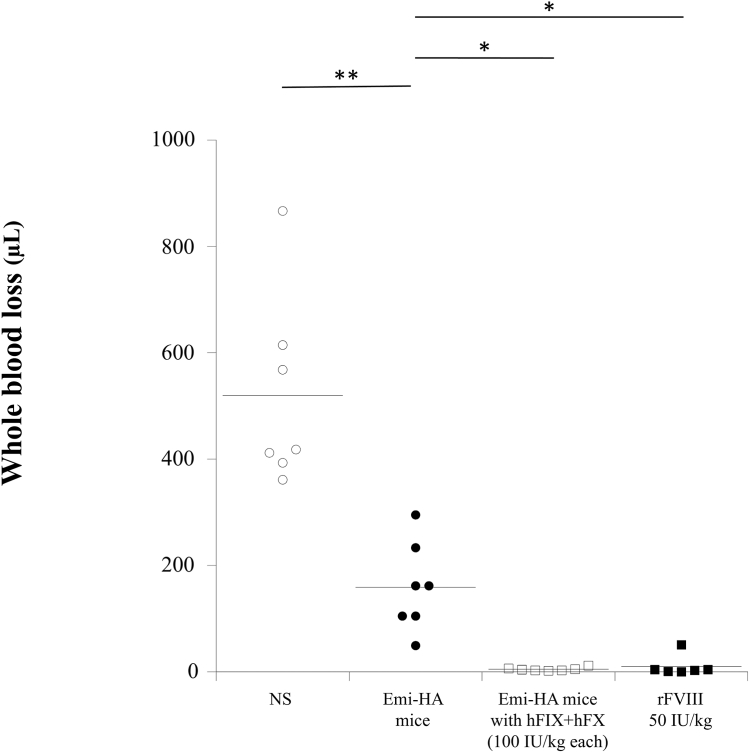
*In vivo* hemostatic effects of emicizumab and 2 types of doses of human (h) factor (F)IX and FX in HA mice determined by tail-clip assays. Emicizumab (3 mg/kg), hFIX (100 IU/kg), and hFX (100 IU/kg) were administered to the HA mice (termed Emi-HA mice). Emi-HA mice were administered hFIX and hFX at 100 or 200 IU/kg each. The recombinant (r)FVIII preparation (rurioctocog alfa at 50 IU/kg) was administered to HA mice. The terminal 5 mm of the tail was amputated 5 minutes after administration, and shed blood was collected for 10 minutes as described in the Methods section. Each data point represents a single mouse. The straight line represents the mean values. Statistical analyses between Emi-HA mice (*n* = 7) and other HA mice (*n* = 6-7) were performed using Dunnett multiple comparison test. Significant differences were defined as *P* < 0.05 (∗*P* < .05; ∗∗*P* < .01). Emi, emicizumab; HA, hemophilia A; NS, normal saline.

The hemostatic efficacy of emicizumab-HA mice was evaluated using the tail-clip assay, as described in the Methods section [19]. The volume of blood loss in the emicizumab-HA mice was lower than that in the naive HA mice. Moreover, the volume of blood loss in rFVIII-administered HA mice and emicizumab-HA mice with additional hFIX/hFX (100 IU/kg each) was significantly reduced relative to that in emicizumab-HA mice (Figure 5; Supplementary Table 2). This result supported the validity of the ROTEM analyses and confirmed that additional supplementation with hFIX/hFX (100 IU/kg each) further augmented the hemostatic effects of emicizumab in HA mice.

### 3.5 Evaluation of thrombotic state in emicizumab-HA mice with additional hFIX and hFX

To investigate the thrombotic state in the emicizumab-HA mice and those treated with additional hFIX/hFX (100 IU/kg each), we measured their TAT complexes and D-dimer levels. The TAT complexes and D-dimer values were not significantly different between the 2 conditions in HA mice (Table 2), indicating that additional hFIX and hFX were unlikely to increase the thrombotic risk in emicizumab-HA mice.

**Table 2:** The D-dimer and TAT complexes in HA mice administered with emicizumab, hFIX, and FX.

Parameters	HA mice	HA mice with Emi (3 mg/kg) + hFIX/hFX (100 IU/kg each)	HA mice with Emi (3 mg/kg) + hFIX/hFX (100 IU/kg each) + additional hFIX/hFX (100 IU/kg each)	WT mice
D-dimer (ng/mL)	397 ± 74	338 ± 59	331 ± 33	379 ± 45
TAT complexes (ng/mL)	0.8 ± 0.5	0.6 ± 0.3	0.7 ± 0.2	0.8 ± 0.3

HA mice were administered with emicizumab (3 mg/kg); (h)FIX and hFX (100 IU/kg each) or emicizumab (3 mg/kg); and hFIX and hFX (100 IU/kg each) with additional hFIX and hFX (100 IU/kg each). The D-dimer and TAT complexes were measured in HA mice and WT mice (*n* = 5-6 each). The means and SDs of the parameters are shown. Comparison of these parameters between native HA mice and other mice was performed using the Dunnett test. No significant differences were detected among them.

Emi, emicizumab; F, factor; h, human; HA, hemophilia A; TAT, thrombin–antithrombin; WT, wild-type.

### 3.6 Comparison of the concentrations of FIX-emicizumab-FX ternary complexes in emicizumab-treated persons with HA with additional FIX and FX

**Figure 6:**
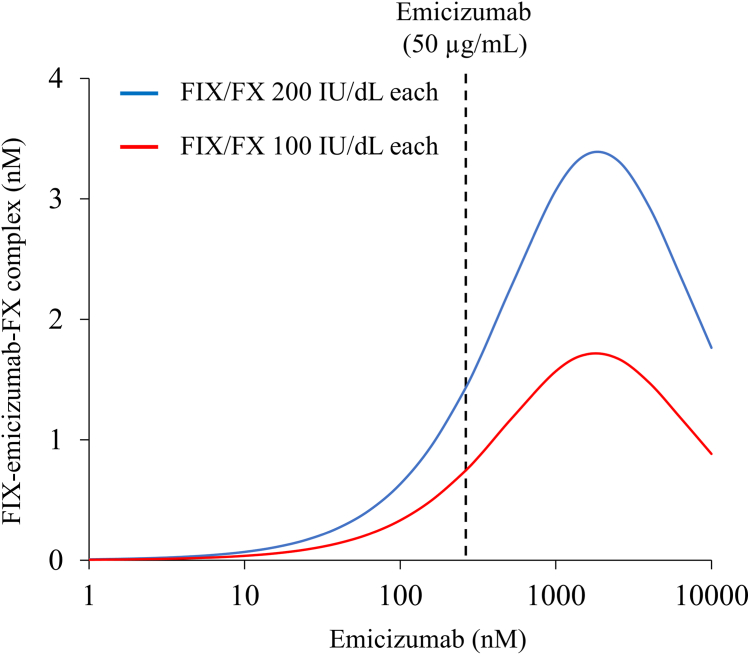
Simulated prediction of the concentrations of factor (F)IX-emicizumab-FX ternary complexes in emicizumab-treated persons with HA with additional FIX and FX. The estimated concentrations of the FIX-emicizumab-FX ternary complexes are shown: red, normal FIX:C (100%) and FX:C (100%); blue, high concentrations of FIX:C (200%) and FX:C (200%). The dashed line represents the FIX-emicizumab-FX ternary complex under the presence of emicizumab (50 μg/mL).

We hypothesized that the enhanced coagulation function in emicizumab with FIX/FX 200 IU/dL may be due to the increase of the FIX-emicizumab-FX ternary complexes. Therefore, we simulated the kinetics of FIX-emicizumab-FX ternary complexes in persons with HA receiving emicizumab with additional FIX and FX (Figure 6). The generation of FIX-emicizumab-FX ternary complexes in emicizumab-treated persons with HA with additional FIX and FX (100 IU/dL each) was approximately 2-fold higher than that in those without additional FIX/FX (Figure 6), supporting that the enhancement of coagulation potential observed with emicizumab in the presence of FIX/FX at 200 IU/dL may be attributed to an increased formation of the FIX-emicizumab-FX complex.

## 4 Discussion

In persons with HA and inhibitors receiving emicizumab, rFVIIa is typically used as first-line treatment [33]. However, we have previously reported that repeated administration of rFVIIa leads to FX consumption, possibly reducing its hemostatic efficacy [15]. This suggests that the efficacy of rFVIIa diminishes over time, highlighting the need for alternative therapeutic strategies. More recently, it was reported that emicizumab may be a potential therapeutic option for selected persons with hemophilia B [16], supporting the notion that formation of the FIX-emicizumab-FX complex, even in the presence of small amounts of FIX/FIXa, could enhance coagulation potential. We also demonstrated that FX supplementation augments the emicizumab-driven coagulation potential [19]. Based on these findings, we hypothesized that the addition of small amounts of both FIX and FX further enhances the hemostatic effect of emicizumab; our present experimental results support this hypothesis.

Our simulated results showed that the generation of FIX-emicizumab-FX ternary complexes in emicizumab-treated persons with HA with additional FIX and FX (100 IU/dL each) was approximately 2-fold higher than that in those without additional FIX/FX (Figure 6), suggesting that an approximately 2-fold increase could be associated with enhanced emicizumab-driven coagulation potential to near-normal levels. Conversely, we previously demonstrated that a reduction in the formation of the FIX-emicizumab-FX complex led to decreased coagulation potential in emicizumab-treated persons with HA [34], supporting the importance of generating FIX-emicizumab-FX complexes in the emicizumab-treated state. If the plasma concentrations of FIX and FX increase at high emicizumab concentrations, ternary complex formation should increase. Therefore, careful consideration of the emicizumab concentration is warranted.

Regarding the data of emicizumab-treated persons with HA with inhibitors, case 2 exhibited a high thrombin generation potential with emicizumab treatment alone (Figure 3). We speculate that this enhanced coagulation potential may be due to an increased amount of FXa generated through the emicizumab-FIXa-FX complex. In such cases, the coagulant effect may be limited even though additional 100 IU/dL of both FIX and FX are supplemented because of a modest increase in FXa generation. As for the ROTEM analysis, the reason(s) why the CT + CFT results for 3 patients are quite different remains unclear. The different CT + CFT results for them may not be associated with emicizumab concentration because their emicizumab concentration was equivalent (41, 32, and 37 μg/mL, respectively). Whole blood samples contain platelets, and previous study demonstrated that emicizumab promotes FXa generation on endothelial cells [35]. In this context, we considered that the amount of FXa generated from emicizumab-FIXa-FX complex on endothelial cells or platelet surfaces may exhibit interindividual variability.

When FIX/FX or rFVIIa was added to the plasma of emicizumab-treated persons with HA with inhibitors, the coagulation potential was nearly comparable, as demonstrated by CWA and TGA. These findings suggest that supplementation with both FIX and FX may serve as an alternative hemostatic therapy for emicizumab-treated persons with HA with inhibitors. The half-lives of rFVIIa, FIX, and FX in blood circulation are reported as approximately 2.5 [36], 24 [37], and 40 hours [38], respectively. Therefore, less frequent administration of FIX and FX may be suitable for the hemostatic management of these patients. From the perspective of monitoring, an earlier investigation indicated that emicizumab activity could be assessed using a human chromogenic substrate assay and surrogate FVIII:C activity could be speculated [39]. Supplementation with both FIX and FX in the presence of emicizumab may be useful for measuring the coagulation function using human chromogenic substrate assay. In addition, FVIII inhibitors may develop when FVIII concentrates are administered to patients with severe persons with HA. As persons with nonsevere HA show a low level of FVIII activity [40], the likelihood of developing FVIII inhibitors was considerably lower in these groups [41]. Therefore, since sufficient levels of FIX and FX exist in these patients, the risk of developing FIX and FX inhibitors may be considered low.

Our TGA results (Table 1) exhibited that the ETP value of FVIII-deficient plasma mixed with emicizumab and FIX/FX (200 IU/dL each) was not different from that of FVIII-deficient plasma mixed with emicizumab and FIX/FX (100 IU/dL each). When these results and the lack of ETP improvement are interpreted in the context of clinical settings, the maximum coagulation potential of emicizumab with FIX/FX 200 IU/dL each appears to be comparable with the normal range; however, its duration of hemostatic efficacy may be reduced compared with rFVIII. Further studies are warranted to clarify this point.

Our tail-clip assay results showed that blood loss volume of emicizumab with FIX/FX 200 IU/kg was significantly lower than that of emicizumab with FIX/FX 100 IU/kg each and similar to that of rFVIII in a short tail clip assay (10 minutes). In contrast, blood loss of emicizumab with FIX/FX 200 IU/kg each was not different from that of emicizumab with FIX/FX 100 IU/kg each but was significantly higher than that of rFVIII in a longer tail clip assay at 30 minutes (Supplementary Figure 1; Supplementary Table 2). These results indicate that the clots formed with FVIII and those formed with emicizumab in the presence of FIX/FX (200 IU/kg each) were not identical and that those formed with rFVIII were more stable than those generated with emicizumab in the presence of FIX/FX (200 IU/kg each). The reason(s) why the result of a tail-clip experiment between 10 and 30 minutes are different remains unclear; however, we speculate that the difference may be associated with the shorter half-life of human FXa and the clot instability in murine circulation. Previous study demonstrated that clots formed by emicizumab are less stable compared with those formed by FVIII [42]. As for the half-life of human FX, it is generally known that the half-life of human FX in human blood circulation is approximately 40 hours [38]. In contrast, recent report suggested that the half-life of human FX in murine circulation is approximately 5 hours [43], indicating that the half-life of human FX in murine circulation resulted in approximately one-eighth relative to that in human circulation. Similarly, we consider that the half-life of human FXa generated from the emicizumab-FIXa-FX complex in murine circulation may be markedly shorter than that in humans. Overall, although the amount of FXa generated with emicizumab in the presence of FIX/FX 200 IU/kg each was greater than that in the presence of FIX/FX 100 IU/kg each, the rapid clearance of FXa and clot instability in murine circulation may lead to a different blood loss of tail-clip assay between 10 and 30 minutes.

Some nonfactor products such as emicizumab, concizumab, and fitusiran have been developed [44]. However, thrombotic complications associated with the use of these nonfactor therapies have been reported. Thrombotic events under emicizumab prophylaxis were recorded during repeated concomitant APCC treatments [11]. Sinus vein thrombosis was observed in noninhibitor persons with HA treated with a combination of fitusiran and FVIII concentrates [45]. In terms of concizumab, 2 arterial and 3 venous thrombotic events have been reported in 3 persons with HA with inhibitors [46]. These findings highlight the need for careful monitoring of the thrombotic risk in persons with HA receiving nonfactor therapies. A previous report documented elevated D-dimer and prothrombin fragments 1 and 2 levels in fitusiran-treated persons with HA in 2 cases of thrombotic events [47]. In contrast, our experimental results showed that the TAT and D-dimer levels in emicizumab-HA mice with additional hFIX/hFX (100 IU/kg each) were comparable with those in mice without additional hFIX/hFX. Although further investigations are warranted to confirm the thrombotic risk, these results support the notion that thrombotic risk may not increase.

There are some limitations in the present study. We could not conduct spiking experiments with anti-emicizumab anti-idiotype antibodies as negative controls using patients’ blood samples because of limited volumes of patients’ blood samples. Nevertheless, we demonstrated that the combined administration of exogenous FIX and FX may serve as an effective hemostatic strategy for managing breakthrough bleeding in emicizumab-treated persons with HA with inhibitors. Further studies are underway to validate the feasibility and efficacy of concomitant FIX and FX supplementation in emicizumab-treated persons with HA.

## Figures and Tables

**Supplementary Figure:**