# Supplementary material for: Augmented emicizumab-driven coagulation potential in hemophilia A state by in vitro and in vivo supplementation of combined factors IX and X
Source: Res Pract Thromb Haemost. 2025 Dec 30;10(1):103329. doi: 10.1016/j.rpth.2025.103329 (PMC12856453; doi:10.1016/j.rpth.2025.103329)
Supplement: Supplementary Table [file mmc1.docx]

**Supplemental Table 1. *In vitro* parameters determined by global coagulation assays in FVIII-deficient plasmas after addition of various amounts of exogenous FIX and FX 100 IU/dL.**

Clot waveform assay (CWA) and thrombin generation assay (TGA) in FVIII-deficient plasmas spiked with various amounts of exogenous FIX concentrates and with/without FX 100 IU/dL were performed as described in the Material and Methods. Two different batches of FVIII-deficient plasma were examined twice for each batch, and the mean and standard deviation of the parameters are shown. CWA and TGA parameters obtained from FVIII-deficient plasmas alone were used as the control group and were compared with those from FVIII-deficient plasmas spiked with FIX and/or FX. No significant differences were detected by the Dunnett’s test. The Ad|min1| value obtained from healthy individuals was 7.2±0.6, and the peak thrombin and ETP values obtained from healthy individuals were 215±37 nM and 2,882±306 nM×min, respectively. Abbreviation: FVIII-def; FVIII-deficient plasma, Ad|min1|; adjusted |min1|, PeakTh; peak thrombin, FIX 100, 200, 400, 800, 1600; FIX 100, 200, 400, 800, 1600 IU/dL, respectively. FX 100; FX 100 IU/dL.

|  | **Parameters** | **FVIII-def** | **No Emicizumab** | | | | | | | | | |
| --- | --- | --- | --- | --- | --- | --- | --- | --- | --- | --- | --- | --- |
|  |  |  | **No FX** | | | | | **FX 100** | | | | |
|  |  |  | **FIX 100** | **FIX 200** | **FIX 400** | **FIX 800** | **FIX 1600** | **FIX 100** | **FIX 200** | **FIX 400** | **FIX 800** | **FIX 1600** |
| CWA | Ad\|min1\| | 3.6 ± 0.2 | 3.5 ± 0.1 | 3.4 ± 0.1 | 3.4 ± 0.1 | 3.2 ± 0.1 | 2.9 ± 0.1 | 4.1 ± 0.3 | 4.1 ± 0.3 | 4.1 ± 0.3 | 4.1 ± 0.4 | 3.9 ± 0.4 |
| TGA | PeakTh (nM) | 106 ± 27 | 107 ± 36 | 104 ± 31 | 98 ± 26 | 90 ± 20 | 83 ± 21 | 178 ± 38 | 173 ± 33 | 171 ± 29 | 163 ± 24 | 159 ± 26 |
|  | ETP (nM×min) | 2624 ± 311 | 2772 ± 570 | 2693 ± 543 | 2499 ±  382 | 2378 ± 273 | 2223 ± 384 | 2968 ± 124 | 3012 ± 240 | 3083 ± 409 | 2955 ± 230 | 2946 ± 336 |

**Supplemental Table 2. Bleeding profile of emicizumab and two types of doses of human FIX and FX in HA mice determined by tail-clip assay**

Emicizumab (3 mg/kg), human (h)FIX (100 IU/kg), and hFX (100 IU/kg) were administered to the HA mice (termed Emi-HA mice). Emi-HA mice were administered hFIX and hFX at 100 IU/kg each, or a combined total of 200 IU/kg each. The rFVIII preparation (rurioctocog alfa at 50 IU/kg) was administered to HA mice. The terminal 5 mm of the tail was amputated 5 min after administration, and shed blood was collected for 10 or 30 min as described in the Methods section. The means and standard deviations of the blood loss volume are shown. Statistical analyses between Emi-HA mice and other HA mice (10 and 30 min, respectively) were performed using Dunnett’s multiple comparison test. Significant differences were defined as p <0.05 (*p <0.05, **p <0.01). hFIX, human FIX; hFX, human FX; NS, normal saline.

|  | NS | Emi-HA mice | Emi-HA mice with hFIX+hFX (100 IU/kg each) | rFVIII 50 IU/kg |
| --- | --- | --- | --- | --- |
| Blood loss volume at 10 min (µL) | 519 ± 180** | 159 ± 84 | 5 ± 3* | 11 ± 2* |
| Blood loss volume at 30 min (µL) | 836 ± 72* | 557 ± 218 | 456 ± 152 | 20 ± 3** |

**Supplementary data**

**Supplementary Figure 1. *In vivo* hemostatic effects of emicizumab and two types of doses of human FIX and FX in HA mice determined by longer tail-clip assays.**

Emicizumab (3 mg/kg), human (h)FIX (100 IU/kg), and hFX (100 IU/kg) were administered to the HA mice (termed Emi-HA mice). Emi-HA mice were administered hFIX and hFX at 100 IU/kg each or 200 IU/kg each. The rFVIII preparation (rurioctocog alfa at 50 IU/kg) was administered to HA mice. The terminal 5 mm of the tail was amputated 5 min after administration, and shed blood was collected for 30 min as described in the Methods section. Each data point represents a single mouse. The *straight line* represents the mean values. Statistical analyses between Emi-HA mice (n=5) and other HA mice (n=4-5) were performed using Dunnett’s multiple comparison test. Significant differences were defined as p <0.05 (*p <0.05, **p <0.01). hFIX, human FIX; hFX, human FX; NS, normal saline.
